# Safety profile of rivaroxaban in first-time users treated for venous thromboembolism in four European countries

**DOI:** 10.1371/journal.pone.0298596

**Published:** 2024-03-07

**Authors:** Ana Ruigómez, Tania Schink, Annemarie Voss, Ron M. C. Herings, Elisabeth Smits, Karin Swart-Polinder, Yanina Balabanova, Gunnar Brobert, Kiliana Suzart-Woischnik, Luis Alberto García Rodríguez

**Affiliations:** 1 Spanish Centre for Pharmacoepidemiological Research (CEIFE), Madrid, Spain; 2 Leibniz Institute for Prevention Research and Epidemiology–BIPS, Bremen, Germany; 3 PHARMO Institute for Drug Outcomes Research, Utrecht, Netherlands; 4 Bayer AG, Berlin, Germany; 5 Bayer AB, Stockholm, Sweden; Kurume University School of Medicine, JAPAN

## Abstract

**Background:**

The European rivaroxaban post-authorization safety study evaluated bleeding risk among patients initiated on rivaroxaban or vitamin K antagonists for the treatment and secondary prevention of venous thromboembolism in routine clinical practice.

**Methods:**

Cohorts were created using electronic healthcare databases from the UK, the Netherlands, Germany and Sweden. Patients with a first prescription of rivaroxaban or vitamin K antagonist during the period from December 2011 (in the UK, January 2012) to December 2017 (in Germany, December 2016) for venous thromboembolism indication, with no record of atrial fibrillation or recent cancer history, were observed until the occurrence of each safety outcome (hospitalization for intracranial, gastrointestinal, urogenital or other bleeding), death or study end (December 2018; in Germany, December 2017). Crude incidence rates of each outcome per 100 person-years were computed.

**Results:**

Overall, 44 737 rivaroxaban and 45 842 vitamin K antagonist patients were enrolled, mean age, 59.9–63.8 years. Incidence rates were similar between rivaroxaban and vitamin K antagonist users with some exceptions, including higher incidence rates for gastrointestinal bleeding in rivaroxaban users than in vitamin K antagonist users. Among rivaroxaban users, mortality and bleeding risk generally increased with age, renal impairment and diabetes.

**Conclusions:**

This study provides further data from routine clinical practice that broadly support safety profile of rivaroxaban for VTE indication and complement findings from previous randomized clinical trials.

## Introduction

Venous thromboembolism (VTE), clinically presenting as deep vein thrombosis (DVT) or pulmonary embolism (PE), is associated with significant morbidity and mortality. Epidemiological modelling for six European countries estimated nearly 762 000 symptomatic VTE events/year and over 370 000 VTE-related deaths/year [1]. Risk factors for VTE (other than major surgery and trauma) [2] include increasing age [3], cancer [3], hospitalization [4] and previous history of VTE [5].

Anticoagulants are the cornerstone of treatment and secondary prevention of VTE [3, 6]. The choice of anticoagulant is driven by patient characteristics, including comorbidities and comedications, and practice guidelines for the relevant healthcare system [3, 6]. The primary safety concern for all anticoagulants is the increased risk of bleeding, especially events requiring hospitalization. Understanding the likely bleeding risk for a patient is a key component of the benefit–risk evaluation of anticoagulant treatment when deciding on a drug and treatment duration.

Rivaroxaban is an oral, direct, factor Xa inhibitor that has been approved for several indications, including the treatment and secondary prevention of VTE in adults [7, 8]. In clinical trials in patients with VTE, rivaroxaban was associated with a lower rate of major bleeding than the standard of care (enoxaparin and either warfarin or acenocoumarol) [9]. Shortly after the first approval of rivaroxaban in Europe in 2011, a pharmacoepidemiological post-authorization safety study (PASS) programme was initiated to monitor patterns of rivaroxaban use, patient characteristics and safety and effectiveness outcomes in first-time users of rivaroxaban and vitamin K antagonists (VKAs) treated for the condition in routine clinical practice [10]. One element of the rivaroxaban PASS incorporates four observational cohort studies, collectively covering the UK, the Netherlands, Germany and Sweden over 5 years.

The objective of the present study was to investigate the safety outcomes (intracranial, gastrointestinal, urogenital and other bleeding events leading to hospitalization), selected based on the outcomes included in pivotal randomized controlled trials (RCTs) of rivaroxaban [11, 12]. Additionally, we evaluated mortality risk and bleeding outcomes according to old age, renal function and diabetes among rivaroxaban users.

## Methods

### Study design

The study design of this PASS has been previously published in detail [10]. Study cohorts were created using healthcare databases from four European countries. Enrolment of patients aged 2 years or older who were first-time users of rivaroxaban or VKA for an indication of the treatment and secondary prevention of VTE began on 9 December 2011 (Germany, Netherlands and Sweden) or 1 January 2012 (UK) and continued until 31 December 2016 (Germany) or 31 December 2017 (UK, Netherlands and Sweden); the observation period ended 1 year after the end of the enrolment period. Patients who previously received treatment with other forms of anticoagulant therapy were eligible for inclusion (S1 Table).

The start date for each patient was defined as the date of first prescription/dispensation of study drug (rivaroxaban or VKA) during the enrolment period. The comparator study drug was defined as the most widely used VKA(s) in each of the respective countries (UK and Sweden, warfarin; Germany, phenprocoumon; Netherlands, acenocoumarol or phenprocoumon). An indication of the treatment and secondary prevention of VTE was assigned when a diagnostic code for DVT and/or PE was recorded close to the prescription date (3 months prior), in the absence of an atrial fibrillation code at any time point.

VKA users were included in the PASS to provide contemporary context for rivaroxaban use because VKAs were the standard of care at the time of study initiation. The study was not designed for direct statistical comparisons of incidence rates (IRs) among anticoagulants or countries and therefore only absolute risks are presented. Patients with a recent history of cancer (i.e. a cancer code received from 3 years before to 1 month after the start date was used as a proxy for active cancer) were excluded from the present analyses because patients with active cancer are a heterogeneous group that warrants specific consideration owing to a variably increased risk of thromboembolic and bleeding events, which makes the choice of anticoagulant and duration of treatment particularly challenging [13–15].

### Data sources

The data sources for the PASS included the following: the IQVIA Medical Research Data (IMRD), incorporating The Health Improvement Network(THIN), a Cegedim database; the German Pharmacoepidemiological Research Database (GePaRD); the PHARMO Database Network, Netherlands (for the general practitioner sub-cohort in this analysis, data were extracted from the Out-patient Pharmacy Database, Hospitalisation Database and General Practitioner Database); and Swedish nationwide health registries [10]. Approval of individual study protocols were granted by the appropriate research ethics committees and regulatory authorities (S2 Table). The four studies are registered separately on the ENCePP (European Network of Centres for Pharmacoepidemiology and Pharmacovigilance) electronic register (EUPAS11299, EUPAS11141, EUPAS11145, and EUPAS9895) and the ClinicalTrials.gov website (NCT01947998, NCT01947985, NCT01947959, and NCT02468102).

### Outcomes

The definitions of outcomes were based on specific diagnostic procedure codes (Read Codes in the UK, or International Statistical Classification of Diseases and Related Health Problems [ICD] 9th Revision or ICD 10th Revision in Germany, the Netherlands and Sweden) and were harmonized between databases as much as possible for the purpose of this analysis; studies that detail the design and validation of this methodology have previously been published [10, 16]. The evaluated safety outcomes included bleeding events that led to hospitalization (as measure of severity), categorized as intracranial haemorrhage (intracerebral haemorrhage, subarachnoid haemorrhage or subdural haematoma), gastrointestinal bleeding (upper and lower gastrointestinal tract), urogenital bleeding and bleeding events at other sites [17]. All-cause mortality was also evaluated among rivaroxaban users.

### Statistical methods

The data from each country were analysed separately and anonymously, authors have no access that could identify patients participants. Data were not pooled between countries due to databases differences and to permit transparency of findings. Independent follow-ups were performed for each outcome, and patients were censored at whichever came first of withdrawal from the database; first occurrence of the safety outcome of interest; treatment switch or discontinuation from the initial drug (with a 30-day grace period after the end of supply of the study drug); death or the end of the observation period. The safety outcomes for first-time users of rivaroxaban or VKAs during their first episode of treatment were analysed as unadjusted IRs with 95% confidence intervals (CIs) computed as the number of events per 100 person-years. IRs of bleeding and all-cause mortality were also calculated in specific high-risk subgroups of rivaroxaban users, including patients with impaired renal function, patients aged 75 years or older and patients with type 1 or type 2 diabetes. No comparative statistical analyses of unadjusted IRs of bleeding outcomes between rivaroxaban and VKAs were conducted because these drugs were likely prescribed to groups of patients with different characteristics that cannot be fully adjusted for in the analyses.

## Results

### Study population

The analyses included 44 737 patients treated with rivaroxaban and 45 842 treated with VKAs (mean age, 59.9–63.8 years; approximately 1:1 ratio of male to female patients). Baseline characteristics for patients were broadly similar across the countries (Table 1). The proportion of patients with a history of bleeding across cohorts ranged from 0.5% (intracranial bleeding in VKA-treated patients in Germany) to 11.4% (urogenital bleeding in rivaroxaban-treated patients in the UK). Most patients were naive to oral anticoagulant therapy at the start date (rivaroxaban users, 87.7–98.5%; VKA users, 99.0–99.6%).

**Table 1:** General characteristics of study patients: First-time users of rivaroxaban or VKA for the treatment and secondary prevention of VTE.

	UK		Netherlands		Germany		Sweden	
	Rivaroxaban	VKA(s)	Rivaroxaban	VKA(s)	Rivaroxaban	VKA(s)	Rivaroxaban	VKA(s)
	N = 5680	N = 4636	N = 586	N = 2617	N = 25 914	N = 20 502	N = 12 557	N = 18 087
Sex								
Male	2809 (49.5)	2335 (50.4)	274 (46.8)	1278 (48.8)	11 860 (45.8)	9290 (45.3)	6319 (50.3)	8913 (49.3)
Female	2871 (50.5)	2301 (49.6)	312 (53.2)	1339 (51.2)	14 054 (54.2)	11 212 (54.7)	6238 (49.7)	9174 (50.7)
Age at first prescription/dispensation, years, mean ± SD	62.3 ± 16.5	62.1 ± 16.1	60.7 ± 16.2	59.9 ± 16.2	60.9 ± 17.4	62.4 ± 16.7	62.1 ± 17.2	63.8 ± 17.2
Age group								
≤ 70 years	3468 (61.1)	2931 (63.2)	389 (66.4)	1881 (71.9)	16 147 (62.3)	12 049 (58.8)	7770 (61.9)	10 511 (58.1)
> 70 years	2212 (39.0)	1705 (36.8)	197 (33.6)	736 (28.1)	9767 (37.7)	8453 (41.2)	4787 (38.1)	7576 (41.9)
Naive to OAC at first prescription/dispensation^a^	4980 (87.7)	4618 (99.6)	545 (93.0)	2606 (99.6)	22 809 (88.0)	20 323 (99.1)	12 372 (98.5)	17 911 (99.0)
Number of contacts with GP during the 12 months before the start date								
0–9	1310 (23.1)	1176 (25.4)	496 (84.6)	2252 (86.1)	–^b^	–^b^	11 716 (93.3)^c^	16 837 (93.1)^c^
10–19	1924 (33.9)	1715 (37.0)	79 (13.5)	317 (12.1)	–^b^	–^b^	715 (5.7)^c^	1033 (5.7)^c^
≥ 20	2446 (43.1)	1745 (37.6)	11 (1.9)	48 (1.8)	–^b^	–^b^	126 (1.0)^c^	217 (1.2)^c^
Number of hospitalizations during the 12 months before the start date								
None	2692 (47.4)	1901 (41.0)	386 (65.9)	1119 (42.8)	6897 (26.6)	4346 (21.2)	5365 (42.7)	5410 (29.9)
1	1361 (24.0)	1224 (26.4)	146 (24.9)	1002 (38.3)	11 984 (46.2)	9875 (48.2)	4660 (37.1)	7669 (42.4)
≥ 2	1627 (28.6)	1511 (32.6)	54 (9.2)	496 (19.0)	7033 (27.1)	6281 (30.6)	2532 (20.2)	5008 (27.7)
Lifestyle characteristics								
Smokers	1124 (19.8)	831 (17.9)	37 (6.3)	182 (7.0)	–^d^	–^d^	244 (1.9)	356 (2.0)
Obesity or BMI > 30	2211 (38.9)	1818 (39.2)	99 (16.9)	341 (13.0)	9194 (35.5)	7473 (36.5)	568 (4.5)	855 (4.7)
Polypharmacy^e^								
≥ 5 medications	3178 (56.0)	2494 (53.8)	239 (40.8)	1421 (54.3)	8395 (32.4)	7317 (35.7)	–^e^	–^e^
Medications of interest prescribed up to 90 days before or on the start date								
Antiplatelets	1068 (18.8)	906 (19.5)	54 (9.2)	254 (9.7)	1295 (5.0)	1135 (5.5)	1204 (9.6)	2242 (12.4)
NSAIDs	825 (14.5)	721 (15.6)	54 (9.2)	192 (7.3)	6756 (26.1)	5147 (25.1)	898 (7.2)	1154 (6.4)
Antidiabetic agents	529 (9.3)	428 (9.2)	38 (6.5)	201 (7.7)	1852 (7.1)	1622 (7.9)	771 (6.1)	1315 (7.3)
Oral steroids	682 (12.0)	551 (11.9)	42 (7.2)	247 (9.4)	2392 (9.2)	2324 (11.3)	1315 (10.5)	2264 (12.5)
PPI	2123 (37.4)	1617 (34.9)	160 (27.3)	846 (32.2)	8670 (33.5)	6985 (34.1)	2122 (16.9)	3413 (18.9)
Medical history: past medical events and comorbidities occurring any time before the start date								
Intracranial bleeding (intracerebral, other)	58 (1.0)	53 (1.1)	4 (0.7)	21 (0.8)	229 (0.9)	104 (0.5)	229 (1.8)	296 (1.6)
Gastrointestinal bleeding	638 (11.2)	470 (10.1)	8 (1.4)	23 (0.9)	447 (1.7)	334 (1.6)	370 (2.9)	544 (3.0)
Urogenital bleeding	649 (11.4)	526 (11.3)	7 (1.2)	24 (0.9)	218 (0.8)	138 (0.7)	662 (5.3)	849 (4.7)
Ischaemic stroke	276 (4.9)	213 (4.6)	9 (1.5)	58 (2.2)	1821 (7.0)	1340 (6.5)	507 (4.0)	915 (5.1)
MI	277 (4.9)	258 (5.6)	35 (6.0)	115 (4.4)	1599 (6.2)	1335 (6.5)	273 (2.2)	566 (3.1)
Hypertension	2260 (39.8)	1900 (41.0)	293 (50.0)	1278 (48.8)	16 325 (63.0)	13 525 (66.0)	3305 (26.3)	5670 (31.3)
Diabetes	770 (13.6)	595 (12.8)	68 (11.6)	276 (10.5)	5566 (21.5)	4738 (23.1)	1054 (8.4)	1823 (10.1)
VTE (DVT/PE)	1287 (22.7)	704 (15.2)	18 (3.1)	9 (< 0.5)	7282 (28.1)	4381 (21.4)	1335 (10.6)	2189 (12.1)
Reduced renal function^f^								
No	3079 (54.2)	2298 (49.6)	215 (36.7)	864 (33.0)	888 (3.4)	675 (3.3)	12 499 (99.5)	17 757 (98.2)
Yes	872 (15.4)	751 (16.2)	57 (9.7)	246 (9.4)	3004 (11.6)	3183 (15.5)	58 (0.5)	330 (1.8)
Unknown	1729 (30.4)	1587 (34.2)	314 (53.6)	1507 (57.6)	22 022 (85.0)	16 644 (81.2)	0 (0.0)	0 (0.0)
Time at risk, first episode of treatment,^g^ days (used in the cohort analyses)								
Mean ± SD	220.9 ± 303.0	296.6 ± 361.9	276.1 ± 209.0	253.5 ± 319.8	330.9 ± 376.7	503.0 ± 555.6	274.0 ± 235.0	562.0 ± 515.0
Median (IQR)	112.0 (42.0–224.0)	190.0 (117.0–274.0)	216.0 (126.0–399.0)	200.0 (80.0–286.0)	207.0 (102.0–412.0)	260.0 (100.0–623.0)	233.0 (145.0–286.0)	333.0 (241.0–723.0)
Total follow-up time,^h^ days (used in the nested case-control analysis)								
Mean ± SD	841.0 ± 497.4	1209.0 ± 707.7	541.1 ± 267.2	1375.8 ± 606.4	1055.5 ± 486.5	1405.7 ± 570.1	1084.0 ± 485.0	1709.0 ± 650.0
Median (IQR)	770.5 (466.0–1196.0)	1191.0 (606.0–1784.0)	515.0 (390.0–672.0)	1367.0 (931.0–1856.0)	1033.0 (664.0–1434.0)	1494.0 (969.0–1896.0)	1086.0 (696.0–1445.0)	1855.0 (1335.0–2218.0)

Data are n (%) unless otherwise stated. Due to rounding, the sum of percentages do not always equal 100%

^a^Patients were categorized as naive or non-naive according to their previous use of any OAC other than their respective study drug any time before the start date

^b^The number of outpatient visits or GP contacts is not assessable in GePaRD

^c^Swedish registers do not contain information on primary care visits. These values are, therefore, open-care visits, which include visits to hospital open clinics, emergency room visits without hospitalization and visits in specialized open care outside hospitals. Data on polypharmacy are not available from these records

^d^Lifestyle information is not included in GePaRD. Obesity was identified by its diagnostic code. Although there is an ICD-10-GM code for heavy smoking, the data are not included here because it is expected that this information is only in the database if the person was treated for this condition

^e^Polypharmacy was defined as the number of medications (based on individual World Health Organization Anatomical Therapeutic Chemical codes) prescribed/dispensed in the 30 days (UK), 3 months (Netherlands) or 1 year (Germany) before the start date. It was not feasible to calculate polypharmacy in the Swedish health registers because information regarding the prescription of drugs were only available for specified medications of interest

^f^Reduced renal function defined as eGFR < 50 mL/min/1.73 m^2^ (UK), eGFR < 60 mL/min/1.73 m^2^ (Netherlands), CKD stages 3–5 diagnostic codes and/or a procedure code for dialysis (Germany) or specific diagnostic codes and/or dispensation of drugs used in renal disease (Sweden)

^g^Until treatment switching/discontinuation, occurrence of the outcome of interest, the end of the study period, death or withdrawal from the database

^h^Until the end of the study period, death or withdrawal from the database

BMI, body mass index; CKD, chronic kidney disease; DVT, deep vein thrombosis; eGFR, estimated glomerular filtration rate; GePaRD, German Pharmacoepidemiological Research Database; GP, general practitioner; ICD-10-GM, International Statistical Classification of Diseases and Related Health Problems 10th Revision–German Modification; IQR, interquartile range; MI, myocardial infarction; NSAID, non-steroidal anti-inflammatory drug; OAC, oral anticoagulant; PE, pulmonary embolism; PPI, proton pump inhibitor; SD, standard deviation; VKA, vitamin K antagonist; VTE, venous thromboembolism

### Incidence of bleeding events

**Fig 1:**
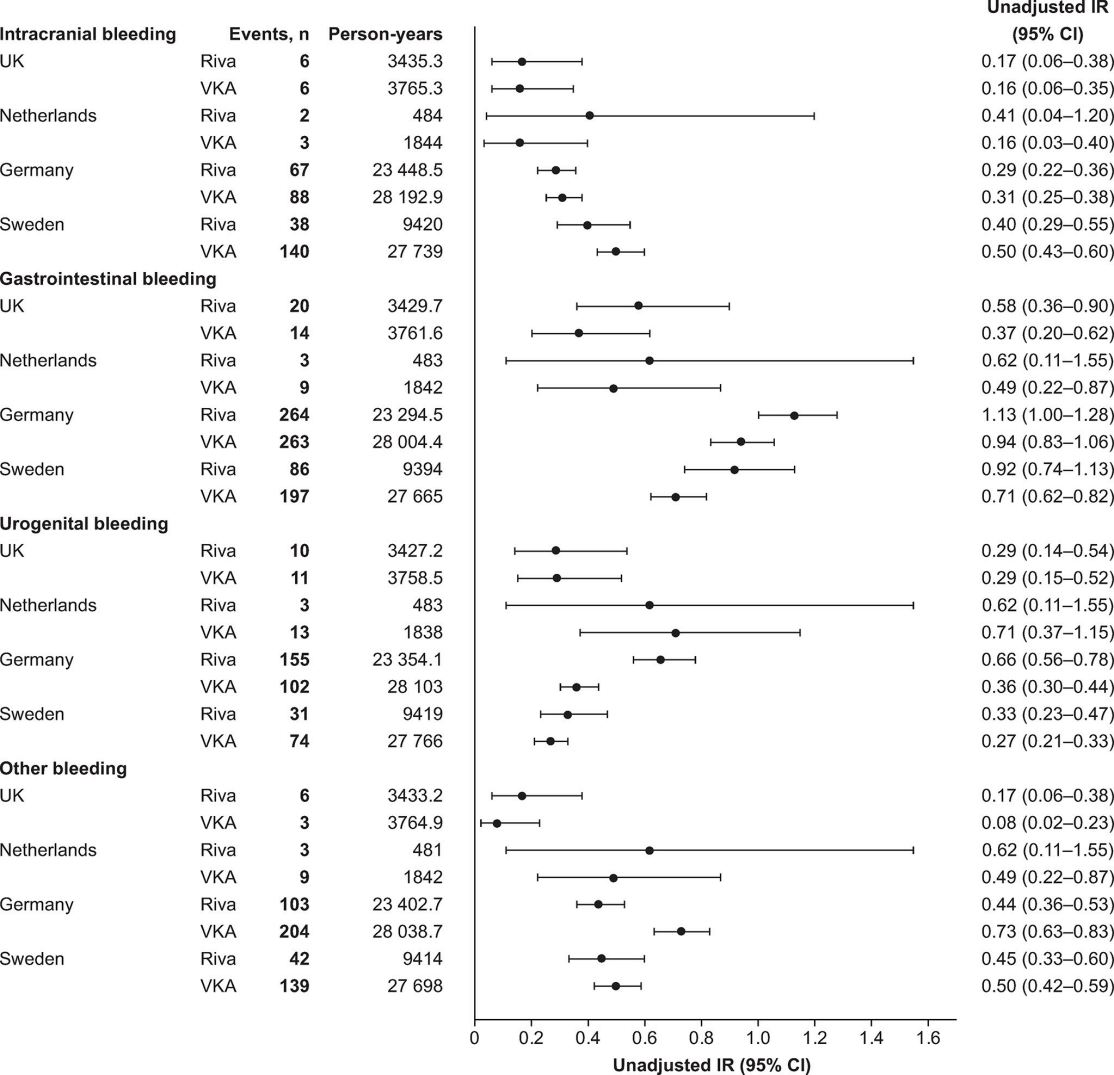
IRs per 100 person-years of safety outcomes associated with first use of rivaroxaban and VKAs for the treatment and secondary prevention of VTE. CI, confidence interval; IR, incidence rate; Riva, rivaroxaban; VKA, vitamin K antagonist; VTE, venous thromboembolism.

The unadjusted IRs for the outcomes of interest are shown in Fig 1. In the UK, Germany and Sweden, intracranial bleeding IRs were similar for the rivaroxaban and VKA groups. The IR for intracranial bleeding was numerically higher for rivaroxaban users than VKA users in the Netherlands, but the 95% CIs overlapped. The urogenital bleeding IR was similar for rivaroxaban and VKA groups in the UK, the Netherlands and Sweden, although in Germany, it was higher in the rivaroxaban group than in the VKA group. In all four countries, gastrointestinal bleeding IRs were numerically higher in patients treated with rivaroxaban than in those treated with VKAs, but CIs overlapped in every comparison. The IR of other bleeding events was similar between treatment groups in the UK, the Netherlands and Sweden but lower in the rivaroxaban group than in the VKA group in Germany.

### Bleeding among specific high-risk subgroups of rivaroxaban users

Among rivaroxaban users in Germany and Sweden, the IRs of intracranial, gastrointestinal and other bleeding leading to hospitalization were all higher for the specific high-risk subgroups of patients than for the corresponding low-risk subgroups (patients with normal renal function, aged < 75 years or with no diabetes), although CIs were often overlapping (Table 2). For patients in Sweden who experienced urogenital bleeding, similar trends were found with respect to age and presence of diabetes. Conversely, urogenital bleeding IRs were higher in the low-risk subgroups than in the high-risk subgroups in Germany with respect to renal function, age and diabetes status. Overall, the results of the subgroup comparisons were more mixed in the UK and the Netherlands, where the numbers of events in rivaroxaban-treated patients were low (Table 2).

**Table 2:** IRs of intracranial, gastrointestinal, urogenital and other bleeding leading to hospitalization associated with first use of rivaroxaban in subgroups of specific interest.

	UK N = 5680			Netherlands^a^ N = 586			Germany N = 25 914			Sweden N = 12 557		
	Events, n	Person-years	IR (95% CI)	Events, n	Person-years	IR (95% CI)	Events, n	Person-years	IR (95% CI)	Events, n	Person-years	IR (95% CI)
**Intracranial bleeding**												
All	6	3435.5	0.17 (0.06–0.38)	2	484	0.41 (0.04–1.2)	67	23 448.5	0.29 (0.22–0.36)	38	9420	0.40 (0.29–0.55)
Normal renal function	5	1852.3	0.27 (0.09–0.63)	1	209	0.5 (0.0–1.9)	53	19 777.3	0.27 (0.20–0.35)	37	9376	0.39 (0.29–0.54)
Impaired renal function	1	603.7	0.17 (0.00–0.92)	0	21	N/A	14	3671.2	0.38 (0.21–0.64)	1	44	2.30 (0.32–16.30)
Age < 75 years	4	2410.0	0.17 (0.05–0.42)	1	365	0.3 (0.0–1.1)	31	16 797.5	0.18 (0.13–0.26)	22	7088	0.31 (0.20–0.47)
Age ≥ 75 years	2	1025.3	0.20 (0.02–0.70)	1	119	0.8 (0.0–3.4)	36	6651.0	0.54 (0.38–0.75)	16	2332	0.69 (0.42–1.12)
No diabetes	6	2942.3	0.20 (0.07–0.44)	2	433	0.5 (0.0–1.3)	48	17 672.1	0.27 (0.20–0.36)	31	8621	0.36 (0.25–0.51)
Type 1 or type 2 diabetes	0	493.0	0.00	0	51	N/A	19	5776.4	0.33 (0.20–0.51)	7	798	0.88 (0.42–1.84)
**Gastrointestinal bleeding**												
All	20	3429.7	0.58 (0.36–0.90)	3	483	0.62 (0.11–1.55)	264	23 294.5	1.13 (1.00–1.28)	86	9394	0.92 (0.74–1.13)
Normal renal function	9^b^	1850.9	0.49 (0.22–0.92)	2	208	1.0 (0.1–2.8)	177	19 690.7	0.90 (0.77–1.04)	83	9351	0.89 (0.72–1.10)
Impaired renal function	9^b^	600.0	1.50 (0.69–2.85)	0	21	N/A	87	3603.8	2.41 (1.93–2.98)	3	43	6.94 (2.24–21.53)
Age < 75 years	9	2407.2	0.37 (0.17–0.71)	1	364	0.3 (0.0–1.1)	106	16 730.7	0.63 (0.52–0.77)	29	7081	0.41 (0.28–0.59)
Age ≥ 75 years	11	1022.6	1.08 (0.54–1.92))	2	119	1.7 (0.1–4.9)	158	6563.8	2.41 (2.05–2.81)	57	2313	2.46 (1.90–3.19)
No diabetes	16	2938.9	0.54 (0.31–0.88)	3	434	0.7 (0.1–1.7)	168	17 569.0	0.96 (0.82–1.11)	72	8602	0.84 (0.66–1.05)
Type 1 or type 2 diabetes	4	490.0	0.81 (0.22–2.09)	0	49	N/A	96	5725.6	1.68 (1.36–2.05)	14	791	1.77 (1.05–2.99)
**Urogenital bleeding**												
All	10	3427.2	0.29 (0.14–0.54)	3	483	0.62 (0.11–1.55)	155	23 354.1	0.66 (0.56–0.78)	31	9419	0.33 (0.23–0.47)
Normal renal function	6^c^	1844.6	0.33 (0.12–0.71)	0	210	N/A	135	19 691.5	0.69 (0.57–0.81)	31	9375	0.33 (0.23–0.47)
Impaired renal function	2^c^	603.7	0.33 (0.04–1.20)	0	21	N/A	20	3662.6	0.55 (0.33–0.84)	0	44	N/A
Age < 75 years	7	2401.8	0.29 (0.12–0.60)	3	363	0.8 (0.1–2.1)	124	16 721.9	0.74 (0.62–0.88)	17	7089	0.24 (0.15–0.39)
Age ≥ 75 years	3	1025.4	0.29 (0.06–0.86)	0	120	N/A	31	6632.2	0.47 (0.32–0.66)	14	2330	0.60 (0.36–1.01)
No diabetes	8	2939.0	0.27 (0.12–0.54)	3	433	0.7 (0.1–1.7)	122	17 601.2	0.69 (0.58–0.83)	22	8622	0.26 (0.17–0.39)
Type 1 or type 2 diabetes	2	488.2	0.41 (0.05–1.48)	0	49	N/A	33	5752.9	0.57 (0.39–0.81)	9	797	1.13 (0.59–2.17)
**Other bleeding**												
All	6	3433.2	0.17 (0.06–0.38)	3	481	0.62 (0.11–1.55)	103	23 402.7	0.44 (0.36–0.53)	42	9414	0.45 (0.33–0.60)
Normal renal function	4	1850.8	0.22 (0.06–0.55)	1	209	0.5 (0.0–1.9)	74	19 745.4	0.37 (0.29–0.47)	41	9371	0.44 (0.32–0.59)
Impaired renal function	2	603.0	0.33 (0.04–1.20)	0	21	N/A	29	3657.3	0.79 (0.53–1.14)	1	43	2.30 (0.32–1.63)
Age < 75 years	3	2408.6	0.12 (0.03–0.36)	2	362	0.6 (0.0–1.6)	57	16 769.7	0.34 (0.26–0.44)	22	7087	0.31 (0.20–0.47)
Age ≥ 75 years	3	1024.6	0.29 (0.06–0.86)	1	119	0.8 (0.0–3.4)	46	6633.1	0.69 (0.51–0.93)	20	2327	0.86 (0.55–1.33)
No diabetes	5	2941.2	0.17 (0.06–0.40)	3	430	0.7 (0.1–1.7)	71	17 648.3	0.40 (0.31–0.51)	38	8617	0.44 (0.32–0.61)
Type 1 or type 2 diabetes	1	492.0	0.20 (0.01–1.13)	0	51	N/A	32	5754.4	0.56 (0.38–0.79)	4	797	0.50 (0.19–1.34)

^a^Patients with unknown renal function were excluded from the analysis of data from the Netherlands, and, therefore, the number of patients from this country with normal renal function and impaired renal function do not always sum to the relevant total in the table

^b^Two UK patients with gastrointestinal bleeding had missing or unknown eGFR values and, therefore, the number of patients from this country with normal renal function and impaired renal function does not sum to the total number of patients with gastrointestinal bleeding

^c^Two UK patients with urogenital bleeding had missing or unknown eGFR values and, therefore, the number of patients from this country with normal renal function and impaired renal function does not sum to the total number of patients with urogenital bleeding

CI, confidence interval; eGFR, estimated glomerular filtration rate; IR, incidence rate; N/A, not available

### Mortality

The IRs of all-cause mortality among rivaroxaban users increased with age and were higher in patients with renal impairment than in those without, and in patients with diabetes than in those without (Table 3). However, the CIs overlapped between treatment groups for all patient subgroups in the Netherlands, and when comparing by diabetes status in the UK.

**Table 3:** IRs of mortality per 100 person-years in first-time users of rivaroxaban (first episode of treatment).

		UK N = 5680			Netherlands^a^ N = 586			Germany N = 25 914			Sweden N = 12 557				
	Events, n		Person-years	IR (95%CI)	Events, n	Person-years	IR (95% CI)	Events, n	Person-years	IR (95% CI)	Events, n		Person-years		IR (95% CI)
**All-cause mortality**															
All rivaroxaban	139		3435.5	4.05 (3.40–4.78)	9	485	1.9 (0.8–3.3)	766	23 473.6	3.26 (3.04–3.50)	304		9431		3.22 (2.88–3.61)
**Analyses by renal function**															
Normal	68^a^		1852.0	3.67 (2.85–4.65)	2^b^	210	1.0 (0.1–2.8)	473	19 794.9	2.39 (2.18–2.61)	297	9387		3.16 (2.82–3.54)	
Impaired	42^a^		604.0	6.95 (5.01–9.40)	2^b^	21	9.7 (0.8–28.3)	293	3678.7	7.96 (7.08–8.93)	7	44		16.06 (7.65–3.68)	
**Analyses by age**															
< 75 years	42		2409.7	1.74 (1.26–2.36)	3	365	0.8 (0.1–2.0)	199	16 812.7	1.18 (1.02–1.36)	106		7095		1.49 (1.23–1.81)
≥ 75 years	97		1025.7	9.46 (7.67–1.54)	6	120	5.0 (1.8–9.9)	567	6660.9	8.51 (7.83–9.24)	198		2336		8.48 (7.37–9.74)
**Analyses by diabetes status**															
No diabetes	109		2942.4	3.70 (3.04–4.47)	8	436	1.8 (0.8–3.4)	470	17 690.7	2.66 (2.42–2.91)	263		8632		3.05 (2.70–3.44)
Type 1 or type 2 diabetes	30		493.0	6.08 (4.11–8.69)	1	49	2.0 (0.0–8.1)	296	5782.9	5.12 (4.55–5.74)	41		799		5.13 (3.78–6.97)

^a^Twenty-nine UK patients who died during the first episode of treatment had missing or unknown eGFR values and, therefore, the number of patients from this country with normal renal function and impaired renal function does not sum to the total number of patients who died

^b^Five Netherlands patients who died during the first episode of treatment had missing or unknown eGFR values and, therefore, the number of patients from this country with normal renal function and impaired renal function does not sum to the total number of patients who died

CI, confidence interval; eGFR, estimated glomerular filtration rate; IR incidence rate

## Discussion

As part of the rivaroxaban PASS programme [10], data from routine clinical practice in four European countries were used to assess risk of bleeding in patients treated for VTE with rivaroxaban or VKA. The safety profile of rivaroxaban in the PASS was consistent with previously reported profiles; no new safety concerns were identified. Unadjusted IRs of safety outcomes among rivaroxaban users for the treatment and secondary prevention of VTE were generally in line with the cumulative incidences reported for the EINSTEIN-DVT and EINSTEIN-PE RCTs and for other real-world studies [11, 12, 18–20].

This PASS was not designed for direct statistical IR comparisons between treatment groups and, because most CIs were overlapping, it was difficult to ascertain trends for consistent differences across countries. Despite this, although both RCTs and real-world data generally showed a similar risk of major bleeding associated with rivaroxaban use compared with VKA use for intracranial and urogenital bleeding [21–25], there is some evidence in the PASS cohorts of increased IRs of gastrointestinal bleeds leading to hospitalization in patients receiving rivaroxaban than in patients receiving VKAs. This is consistent with a meta-analysis that showed increased risk of gastrointestinal bleeding following treatment with direct oral anticoagulants compared with warfarin across indications [26] and also in line with studies on AF patients [27]. However, this was not consistent with other studies in VTE that favoured rivaroxaban over VKAs with respect to gastrointestinal bleeding [22, 23].

Many of the reported observational studies in VTE have follow-up periods of 6 months or less, do not separate major bleeding by site, or do not report IRs by patient-years, thereby making comparisons with the findings reported in the present study difficult. A pooled analysis of the prospective, non-interventional XALIA and XALIA-LEA studies across 36 countries observed patients for more than 6 months and reported unadjusted IRs (95% CI) of any major bleeding of 1.74 (1.24–2.38) per 100 patient-years for rivaroxaban and 3.9 (3.03–5.05) per 100 patient-years for VKAs [20]. In the DRESDEN registry, the IR (95% CI) of major bleeding was 4.1 (2.5–6.4) per 100 patient-years for patients with VTE (median treatment duration, 274 days) [18]. In comparison, the rate of major bleeding in a UK cohort observed for only 12 weeks was higher than in the aforementioned studies, as the cumulative IR (95% CI) of major bleeding with rivaroxaban for the treatment and secondary prevention of VTE was 8.3 (5.3–12.5) [19]. Interestingly, as in previous studies, the results from the PASS also showed IR variations by country. There are likely to be numerous reasons for this observation, including the diversity of patient characteristics from each country and the known differences between healthcare systems. For example, differences in the speed in which each healthcare system recommended the use of rivaroxaban for an indication of VTE inevitably resulted in discrepancies in rivaroxaban uptake compared with VKA uptake in each country.

Consistent with known risk factors and clinical trials [3, 9], our data showed that IRs of intracranial, gastrointestinal and other bleeding events were higher in patients aged 75 years or older. Older age was also found to be an independent risk factor for major bleeding in a study of Swedish national registries (normalized hazard ratio [95% CI]: 1.38 [1.27–1.50]) [28].

In the PASS, the IRs of all-cause mortality were higher in rivaroxaban users aged 75 years or older and in those with impaired renal function or diabetes. The IR (95% CI) of all-cause mortality in this study was nominally higher than that in the pooled analysis of XALIA and XALIA-LEA, which was 1.83 (1.31–2.48) events per 100 patient-years, but many factors could contribute to this difference [20].

Interpretations of the findings must consider the study limitations, some of which are inherent in observational data. The healthcare databases used existed before the study and primarily contained data collected routinely in clinical practice. The objectives, methodologies and codes used to ascertain each outcome and variable were validated and harmonized to the extent feasible between the studies to facilitate descriptive comparisons between countries. However, because of residual differences in the data sources and irreconcilable differences in terms of data availability, no pooled analyses of the studies or statistical comparisons were performed. Nevertheless, although point estimates varied between countries, the direction of trends and magnitudes of effect were broadly similar, except when noted.

Although it is of interest to interpret the results of this PASS in the context of the pivotal RCT findings, neither the background information nor the outcome events are captured in the same manner by these two distinct study approaches. Fundamentally, the patient populations are different; for example, patients were older in the PASS (mean age, 60–64 years) than in the EINSTEIN-DVT and EINSTEIN-PE studies (pooled mean age, 57 years) [9]. However, it is axiomatic that differences in patient characteristics are observed between patients from a real-world study compared with those who have met the strict selection criteria of an RCT. Real-world studies are, therefore, critical in informing benefit–risk assessments in under-represented groups.

## Conclusions

This PASS provides data from routine clinical practice that support the safety profile of rivaroxaban, established from previous RCTs and other post-authorization studies. These findings provide further data to support physicians’ decision-making when prescribing rivaroxaban for VTE.

## Supporting information

S1 Checklist STROBE statement—Checklist of items that should be included in reports of observational studies:”Safety profile of rivaroxaban in first-time users treated for venous thromboembolism in four European countries”. (DOCX)

S1 Table Overview of the individual studies included in the rivaroxaban post-authorization safety study (PASS) program. (DOCX)

S2 Table Characteristics of the data sources used for the study. (DOCX)
