# Supplementary material for: Safety profile of rivaroxaban in first-time users treated for venous thromboembolism in four European countries
Source: PLoS One. 2024 Mar 7;19(3):e0298596. doi: 10.1371/journal.pone.0298596 (PMC10919665; doi:10.1371/journal.pone.0298596)
Supplement: S1 Table — (DOCX) [file pone.0298596.s002.docx]

**Table S1.** Overview of the individual studies included in the rivaroxaban post-authorization safety study (PASS) program.

| Study (country) | Healthcare setting | Study population | Follow-up duration | Safety outcomes | Period of data collection | ENCePP registration |
| --- | --- | --- | --- | --- | --- | --- |
| Database studies | | | | | | |
| IMRD (UK) | Primary care | ≥2–89 years of age, prescribed rivaroxaban or warfarin | 1–7 years | Hospitalization for intracranial, gastrointestinal, urogenital, other bleedings.Death | Dec 2011–Dec 2018 | EUPAS11299 |
| PHARMO (Netherlands) | Primary and secondary care | ≥2–105 years of age, prescribed rivaroxaban or acenocoumarol or phenprocoumon | 1–7 years |  | Dec 2011–Dec 2018 | EUPAS11141 |
| GePaRD (Germany) | Primary and secondary care | ≥2 years of age, prescribed rivaroxaban or phenprocoumon | 1–6 years |  | Dec 2011–Dec 2017 | EUPAS11145 |
| Swedish nationwide health registries (Sweden) | Secondary care | ≥2–108 years of age, prescribed rivaroxaban or warfarin | 1–7 years |  | Dec 2011–Dec 2018 | EUPAS9895 |
