# Supplementary material for: Safety profile of rivaroxaban in first-time users treated for venous thromboembolism in four European countries
Source: PLoS One. 2024 Mar 7;19(3):e0298596. doi: 10.1371/journal.pone.0298596 (PMC10919665; doi:10.1371/journal.pone.0298596)
Supplement: S2 Table — (DOCX) [file pone.0298596.s003.docx]

**Table S2.** Characteristics of the data sources used for the study.

|  | IMRD | PHARMO | GePaRD | Swedish nationwide health registries |
| --- | --- | --- | --- | --- |
| Country | UK | Netherlands | Germany | Sweden |
| Data source | Electronic medical records | Three linked healthcare databases: the Out-patient Pharmacy Database, the Dutch National Medical Register, and the General Practitioner Database | Four statutory health insurance providers | Four linked healthcare registries: the Drug Register, the Patient Register, the Cause of Death Register, and the LISA |
| Size | Data available for more than 3.7 million active patients | Data available for 4 million residents (25% of population) | Data available for ~25 million members (cross-sectionally 17% of population) | Data available for all Swedish residents |
| Representativeness | Representative of the UK population in terms of age, sex, and geographical distribution |  | Coverage of all geographical regions of Germany | Representative of the Swedish population |
| Diagnostic coding systems | Read codes | ICD-9-CM/ICD-10 | ICD-10-GM; EBM; OPS | ICD-10-SE; NOMESCO |
| Specific features | Free text comments may provide additional detail on diagnostic events | Data sources are linked on a patient level through validated algorithms |  | Civic registration number allows individuals to be linked between the different registries |
| Available data lookback period (maximum) | No limit | Overall cohort: 20 years  Sub-cohort with available data in the General Practitioner Database: 10 years | 15 years (since 2004) | Drug Register: 14 years (since 2005)  Patient Register: 22 years (since 1997)  Patient Register (open care): 18 years (since 2001) |

EBM, Einheitlicher Bewertungsmaßstab; GePaRD, German Pharmacoepidemiological Research Database; ICD, International Classification of Diseases; LISA, longitudinal integration database for health insurance and labour market studies; NOMESCO, Nordic Medico-Statistical Committee; OPS, Operations and Procedures Coding System; SOC, standard of care; IMDR (*IQVIA Medical Research Data* incorporating the The Health Improvement Network (THIN), a Cegedin database.
